# Correlation of Neuroimaging Biomarkers and Pharmacogenetic Profiles in Optimizing Personalized Therapy in Children and Adolescents with Psychotic Disorders

**DOI:** 10.3390/neurolint17080128

**Published:** 2025-08-14

**Authors:** Adriana Cojocaru, Adina Braha, Nicoleta Ioana Andreescu, Alexandra Florina Șerban, Codrina Mihaela Levai, Iulius Jugănaru, Iuliana Costea, Lavinia Hogea, Marius Militaru, Iuliana-Anamaria Trăilă, Laura Alexandra Nussbaum

**Affiliations:** 1 Department of Neurosciences, Discipline of Pedopsychiatry, “Victor Babes” University of Medicine and Pharmacy, 2 Eftimie Murgu Square, 300041 Timisoara, Romania; adriana.cojocaru@umft.ro (A.C.); alexandra.sirbu@umft.ro (A.F.Ș.); nussbaum.laura@umft.ro (L.A.N.); 2 Clinical Department of Child and Adolescent Psychiatry, Children’s Emergency Hospital “Louis Turcanu”, “Victor Babes” University of Medicine and Pharmacy, 2 Eftimie Murgu Square, 300041 Timisoara, Romania; 3 Department of Second Internal Medicine—Diabetes, Nutrition, Metabolic Diseases, and Systemic Rheumatology, “Victor Babes” University of Medicine and Pharmacy, 300041 Timisoara, Romania; 4 Department of Microscopic Morphology, Genomic Medicine Centre, “Victor Babes” University of Medicine and Pharmacy, 2 Eftimie Murgu Square, 300041 Timisoara, Romania; andreescu.nicoleta@umft.ro; 5 Doctoral School, “Victor Babes” University of Medicine and Pharmacy, 2 Eftimie Murgu Square, 300041 Timisoara, Romania; 6 Legal Department, “Victor Babes” University of Medicine and Pharmacy, 300041 Timisoara, Romania; codrinalevai@umft.ro; 7 Department XI Pediatrics, Discipline I Pediatrics, “Victor Babes” University of Medicine and Pharmacy, Eftimie Murgu Square 2, 300041 Timisoara, Romania; juganaru.iulius@umft.ro; 8 Psychology Department, West University of Timisoara, 300223 Timisoara, Romania; iuliana.costea@e-uvt.ro; 9 Department of Neurosciences, Neuropsychology and Behavioral Medicine Center, “Victor Babes” University of Medicine and Pharmacy, 2 Eftimie Murgu Square, 300041 Timisoara, Romania; hogea.lavinia@umft.ro; 10 Department of Neurosciences, Discipline of Neurology II, Center of Advanced Research in Cardiology and Hemostaseology, “Victor Babes” University of Medicine and Pharmacy, 300041 Timisoara, Romania; marius.militaru@umft.ro; 11 Department of Microscopic Morphology-Anatomic Pathology, ANAPATMOL Research Center, “Victor Babes” University of Medicine and Pharmacy, 300041 Timisoara, Romania; iuliana.traila@rezident.umft.ro

**Keywords:** pharmacogenetics, neuroimaging biomarkers, personalized psychiatry, pediatric psychotic disorders, CYP2D6 polymorphism

## Abstract

**Background/Objectives:** Psychotic disorders with childhood or adolescent onset pose major therapeutic challenges due to their complex etiology and variable treatment response. While pharmacogenetics and neuroimaging biomarkers have independently shown potential for guiding therapy, their combined utility remains underexplored. This study aimed to investigate whether integrating CYP2D6 pharmacogenetic profiles with structural neuroimaging findings can enhance personalized treatment and predict clinical outcomes in pediatric psychotic disorders. **Methods:** This prospective observational study included 63 children and adolescents (10–18 years) with DSM-5 diagnosed psychotic disorders. All patients underwent baseline MRI and standardized clinical assessments (PANSS, CGI, GAF). CYP2D6 genotyping was performed in 31 patients (49.2%), categorizing them as extensive (EMs) or intermediate metabolizers (IMs). Patients were treated with atypical antipsychotics and followed for 18 months. Outcomes included symptom severity, global functioning, and side-effect profiles. **Results:** EM patients demonstrated superior clinical improvement, as evidenced by a reduction in PANSS scores (from 118 to 40) and a corresponding increase in GAF scores (from 39 to 76), compared to the IM and non-tested groups. IM patients exhibited a higher prevalence of MRI abnormalities and slower response. Significant correlations emerged between CYP2D6 genotype, MRI findings, and treatment outcomes (*p* < 0.001). Combined biomarker profiles enhanced the prediction of therapeutic response and tolerability. **Conclusions:** Integrating CYP2D6 pharmacogenetic data with neuroimaging biomarkers provides valuable guidance for personalizing antipsychotic treatment in pediatric psychosis. This approach improves clinical outcomes and reduces adverse effects. Future research should further explore the integration of multimodal biomarkers in larger, longitudinal cohorts to optimize individualized psychiatric care for this vulnerable population.

## 1. Introduction

Psychotic disorders with onset in childhood and adolescence represent a serious and complex category of mental illnesses. These disorders often debut with hallucinations, delusional thinking, and severe behavioral changes, significantly impacting the developmental trajectory of young patients. Although their prevalence is lower than in adults, early-onset psychoses are more severe, with higher rates of treatment resistance and cognitive impairment [1,2,3]. The multifactorial etiology, encompassing genetic, neurodevelopmental, and environmental influences, demands personalized approaches in both diagnosis and treatment [3,4].

Pharmacogenetics offers the potential to tailor antipsychotic therapy based on an individual’s genetic profile. In particular, polymorphisms in the CYP2D6 gene, which influence the metabolism of many atypical antipsychotics, are clinically significant. Knowing whether a patient is a poor, intermediate, extensive, or ultra-rapid metabolizer can guide drug selection and dosing, minimizing side effects and improving efficacy [5,6,7,8]. In children and adolescents whose metabolic pathways are still developing, such guidance is even more crucial to prevent iatrogenic complications and improve long-term outcomes [5,9].

Structural and functional neuroimaging has revealed numerous brain alterations in youths with psychotic disorders. MRI studies have reported ventricular enlargement, cortical thinning (especially in the frontal and temporal lobes), and volume reductions in subcortical regions such as the hippocampus or amygdala [10,11]. These findings suggest underlying neurodevelopmental disturbances, and their presence may correlate with clinical severity or prognosis. Although widely studied in adult schizophrenia, such biomarkers are less characterized in pediatric populations and are rarely used to guide treatment directly [12,13,14].

Despite the individual utility of pharmacogenetic and neuroimaging data, few studies have explored how these two domains might interact in guiding therapy. For instance, children with both unfavorable neuroanatomical profiles (e.g., cortical thinning) and poor CYP2D6 metabolism may have a higher risk of adverse reactions or insufficient response to standard antipsychotics. Integrating these biomarkers could lead to more precise therapeutic decisions, potentially informing both medication selection and the need for adjunctive interventions [15,16]. The literature supports the hypothesis that combining biological markers increases predictive power for treatment outcomes more than using single parameters alone [17,18].

CYP2D6 metabolizer status reflects how efficiently a person processes certain medications, based on the activity of the CYP2D6 enzyme. Individuals are categorized as follows: poor metabolizers (PMs)—very low or no enzyme activity, with slow drug breakdown and higher risk of side effects; intermediate metabolizers (IMs)—reduced activity, with slower metabolism than normal, and may need dose adjustments; extensive metabolizers (EMs)—normal enzyme activity (typical drug response); and ultra-rapid metabolizers (UMs)—increased enzyme activity (very fast metabolism, may require higher doses or different drugs) [19].

This study aims to explore the associations between CYP2D6 pharmacogenetic profiles, baseline neuroimaging findings, and clinical outcomes in children and adolescents diagnosed with psychotic disorders. Using a cohort analyzed in the doctoral thesis, which includes clinical scales (PANSS, CGI, GAF), adverse event monitoring, and therapeutic evolution, the objective is to determine whether a combined biomarker approach (genetic and imaging) better predicts therapeutic success and guides personalized treatment strategies.

## 2. Materials and Methods

### 2.1. Study Population

This prospective observational study included a cohort of 63 children and adolescents, aged between 10 and 18 years, diagnosed with psychotic disorders according to DSM-5 criteria [20]. The patients were recruited from a tertiary neuropsychiatric hospital in Timișoara, Romania, between 2022 and 2024, with approval from the research ethics committee (No. 32/01.10.2019 rev 2024). Inclusion criteria required the presence of active psychotic symptoms (hallucinations, delusions, disorganized behavior) and parental/legal guardian consent. Patients were newly diagnosed with psychotic disorders and had not received any prior psychotropic treatment, ensuring a treatment-naïve cohort at baseline. Exclusion criteria included psychosis due to medical conditions, substance-induced psychosis, or intellectual disability with an IQ < 70.

Demographic characteristics (age, sex, rural/urban background) and psychiatric and somatic personal and familial history were collected and tabulated. The sample included both treatment-naïve patients and those previously exposed to psychotropic medication.

### 2.2. Clinical and Psychometric Assessment

Patients underwent initial psychiatric evaluation and longitudinal monitoring using standardized scales: the PANSS (Positive and Negative Syndrome Scale) for symptom severity, CGI-S (Clinical Global Impression—Severity) and CGI-I (Improvement), GAF (Global Assessment of Functioning) for global functionality, UKU Side Effect Rating Scale, and AIMS for extrapyramidal symptoms and the Calgary Depression Scale to evaluate affective symptoms. Evaluations were performed at baseline and after 18 months of pharmacological treatment.

The PANSS is one of the most widely used tools to assess symptom severity in schizophrenia spectrum disorders, with a score range of 30–210. It has demonstrated good internal consistency, with Cronbach’s alpha ranging from 0.73 to 0.83 across subscales in various clinical populations [21].

The CGI-S (1–7 score) and CGI-I (1–7 score) scales, though single-item measures, have shown strong inter-rater reliability (ICC values between 0.81 and 0.89) and consistent performance in longitudinal studies involving psychotic disorders [22].

The GAF scale (1–100 score), used to evaluate psychosocial and occupational functioning, has shown acceptable inter-rater reliability, with intraclass correlation coefficients (ICCs) typically reported between 0.61 and 0.86 [22].

The UKU Side Effect Rating Scale assesses a wide range of psychotropic side effects. Its internal consistency has been validated with a Cronbach’s alpha of 0.75 (Lingjærde et al., 1987), indicating acceptable reliability in neuroleptic-treated patients [23].

AIMS, used to detect tardive dyskinesia and other involuntary movements, demonstrates strong reliability, with Cronbach’s alpha values reported between 0.86 and 0.91 [24].

SAS, used to evaluate Parkinsonian symptoms, has shown internal consistency, with a reported Cronbach’s alpha of 0.79 [25].

BARS (1–5 score), which assesses akathisia, reports inter-rater reliability ranging from 0.82 to 0.90, and overall internal consistency with Cronbach’s alpha around 0.86 [26].

The Calgary Depression Scale for Schizophrenia is a validated tool specifically designed to distinguish depressive symptoms from negative or extrapyramidal features in psychotic patients, with scores ranging from 0 to 27. Its Cronbach’s alpha has been reported as 0.83 to 0.92, confirming high internal consistency [27].

### 2.3. Neuroimaging Evaluation

All participants underwent cranial MRI (magnetic resonance imaging) at baseline, using a 1.5 T scanner with standard protocols (T1, T2, FLAIR). Radiological reports assessed structural abnormalities, including sequelae of brain injuries, brain cysts, calcifications of the choroid plexuses and the pineal gland, magna cistern enlargement, and venous angioma. Imaging data were extracted from patient files and categorized into three groups: “normal,” “minor abnormalities,” and “major structural changes.” These were correlated with genotypic profiles and clinical outcomes.

### 2.4. Pharmacogenetic Testing

Genotyping for CYP2D6 polymorphisms was performed using RT-PCR and TaqMan probes, with results interpreted according to CPIC and PharmGKB guidelines. Patients were classified into poor metabolizers (PMs), intermediate metabolizers (IMs), extensive/normal metabolizers (EMs), and ultra-rapid metabolizers (UMs).

In the pharmacogenetic testing protocol, genotyping of CYP2D6 variants was performed using RT-PCR with TaqMan^®^ SNP Genotyping Assays (Applied Biosystems), targeting common alleles relevant for metabolic activity (*3, *4, *5, *6, *10, *17, *41). Primer and probe sequences were pre-validated and provided by the manufacturer. PCR amplification was conducted on a QuantStudio™ 5 Real-Time PCR System, using the following thermal cycling conditions: initial denaturation at 95 °C for 10 min, followed by 40 cycles of 95 °C for 15 s and 60 °C for 1 min.

Quality control measures included the use of non-template controls in each run, and random re-genotyping of 10% of the samples to assess reproducibility, with a concordance rate of 100%. Internal controls and allele-specific positive control DNA were used to verify assay performance and signal discrimination. All genotyping results were reviewed independently by two investigators to ensure consistency.

These results guided subsequent antipsychotic treatment selection or adjustment. Pharmacogenetic results were available for 50.8% of the cohort; the rest received standard treatment protocols without genotypic guidance.

### 2.5. Pharmacological Treatment

All patients received atypical antipsychotic agents, primarily Aripiprazole, Olanzapine, Risperidone, or Quetiapine. Treatment decisions were individualized based on clinical presentation, side-effect profile, and, where available, pharmacogenetic testing. Medication data, including initial and follow-up dosages, switches, and polypharmacy, were recorded in detail.

### 2.6. Statistical Analysis

Statistical analyses were performed using Python Software (version 3.11, Wilmington, DE, USA, 2022; available online: https://www.python.org/ (accessed on 27 June 2025)), utilizing standard libraries such as Pandas, SciPy, and StatsModels for data handling and hypothesis testing. Between-group comparisons involving three groups (EM, IM, and non-tested) were conducted using one-way ANOVA or the Kruskal–Wallis test, depending on data distribution. Correlation analyses examined the relationships between genotype and MRI findings, combined biomarker profiles and symptom improvement (PANSS, CGI), biomarker combinations, and functional outcomes (GAF). Statistical significance was set at *p* < 0.05.

## 3. Results

### 3.1. Distribution of CYP2D6 Genotypes

In this study, pharmacogenetic profiling was conducted to identify the CYP2D6 metabolizer status of patients diagnosed with psychotic disorders (Table 1). This enzyme is crucial in the metabolism of many atypical antipsychotics, affecting both treatment effectiveness and the likelihood of side effects. Of the total group of 63 children and adolescents, pharmacogenetic testing was performed on 31 patients (49.2%). The other 32 patients received standard treatment without pharmacogenetic guidance, serving as a significant comparison group. Participants who did not undergo pharmacogenetic testing were analyzed as a separate “non-tested” group; however, this group may include individuals who, if tested, would have fallen into the PM or UM categories, introducing potential classification bias.

For the entire cohort (*n* = 63), the mean age was 15.22 years (SD ± 1.57), with a median of 15.0 years and an interquartile range of 14.0–17.0; 57.1% were male and 69.8% came from urban areas.

Among the 31 patients who underwent pharmacogenetic testing, the mean age was 15.32 years (SD ± 1.62), and the median was 15.0 years with an interquartile range of 14.0–17.0; 61.3% were male, and 61.3% were from urban environments.

In the total study cohort (*n* = 63), 36 participants (57.1%) were male and 27 (42.9%) were female. Regarding the environment, 44 patients (69.8%) came from urban areas and 19 (30.2%) from rural areas. Among the 31 participants who underwent pharmacogenetic testing, 19 (61.3%) were male and 12 (38.7%) were female. In terms of environment, 19 patients (61.3%) were from urban areas, while 12 (38.7%) were from rural backgrounds.

The results indicate a notable interindividual variability in CYP2D6 enzyme activity among pediatric patients with psychotic disorders. In the tested subgroup, nearly one-third of patients (30.2%) were classified as extensive metabolizers (EMs) and 19% as intermediate metabolizers (IMs). Interestingly, no poor metabolizers (PMs) or ultra-rapid metabolizers (UMs) were identified in this sample.

Beyond pharmacogenetic variability, neuroanatomical alterations may also influence both the clinical presentation and therapeutic response in pediatric psychosis. The following section examines the baseline neuroimaging findings in this cohort and explores their potential relevance in guiding personalized therapeutic strategies.

### 3.2. Neuroimaging Findings and Correlation with Metabolizer Phenotypes

Neuroimaging assessment was performed in all patients at baseline, using structural cerebral MRI (Table 2). The aim was to identify potential brain abnormalities that could inform clinical prognosis and contribute to the development of individualized therapeutic strategies. Overall, 79.37% of the cohort presented regular MRI scans. This is consistent with prior studies, indicating that while gross brain abnormalities are less frequent in pediatric psychotic populations, subtle changes or neurodevelopmental variations may still impact clinical outcomes among the 20.63% of patients with abnormal MRI findings.

The most frequent abnormalities observed were brain cysts (7.94%) and sequelae of prior brain injuries (6.35%). Although these findings are heterogeneous and not specific to psychotic disorders, they may represent markers of altered neurodevelopmental trajectories.

Five cases of cerebral cysts were identified through structural MRI among patients in the pharmacogenetically tested group. The findings were as follows: a small left pericerebellar arachnoid cyst measuring 1.2 × 0.9 cm in the axial plane, non-compressive to adjacent cerebellar structures; a cystic formation with cerebrospinal fluid (CSF) signal measuring 3.3 × 1.8 cm located in the right pontocerebellar and peribulbar cistern, in close relation to the internal auditory canal, associated with right cerebellar hypoplasia; a larger CSF-containing cyst measuring 4.2 × 1.7 cm located anteriorly in the right pontocerebellar region, also accompanied by adjacent cerebellar hypoplasia; a 4.5 mm Rathke’s cleft cyst located in the posterior half of the pituitary gland, showing hypointense signal on both T1 and T2 sequences and demonstrating contrast enhancement; and a 0.7 cm pineal gland cyst.

All patients with these findings underwent neurosurgical consultation. In none of the cases was surgical intervention required, and patients remained under clinical and imaging surveillance. These cystic findings were considered incidental and non-contributory to acute symptomatology but were documented as part of the individualized neuroimaging profile. Notably, rare findings such as venous angioma or calcifications were also identified, albeit in a small subset of the cohort.

The correlation of CYP2D6 metabolizer status and MRI findings revealed distinct patterns across the genotype groups (Table 3). Patients without pharmacogenetic testing (*n* = 32) exhibited a remarkably homogeneous neuroimaging profile, with 100% showing normal MRI findings and no documented structural abnormalities.

Similarly, among the EMs, the vast majority (94.7%) also presented with regular MRI scans, while only one patient in this group exhibited sequelae of prior brain injury. In contrast, the IM group displayed a markedly different profile, characterized by a higher prevalence of MRI abnormalities. Notably, none of the IM patients had a normal MRI. Instead, the majority presented with structural anomalies, including brain cysts (*n* = 5) and sequelae of prior brain injuries (*n* = 3). Moreover, rare findings such as calcifications, magna cistern enlargement, and venous angioma were observed exclusively within this group. The Chi-square test indicates a highly significant association between the CYP2D6 genotype and the presence or type of MRI abnormalities in this cohort (*p*-value < 0.001).

Building on these findings, the following section explores how CYP2D6 genotype and MRI abnormalities jointly influenced clinical outcomes and treatment response in this cohort.

### 3.3. Clinical Evolution Based on Genotype and Neuroimaging

**Figure 1:**
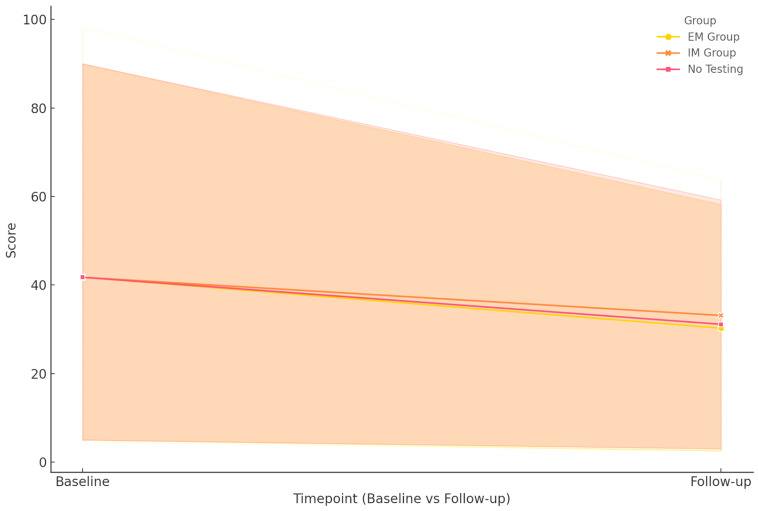
Representation of measured clinical outcomes by genotype.

The evolution of clinical outcomes over the 18-month follow-up period was analyzed by correlating CYP2D6 metabolizer status and MRI findings with changes in symptom severity and global functioning, as measured by the PANSS, CGI-S, CGI-I, and GAF (Table 4 and Figure 1).

PANSS total scores showed a marked and statistically significant decline across the cohort, indicating a reduction in symptom severity. The EM group exhibited the most substantial improvement, with scores dropping from 118 to 40. A similar, albeit less pronounced, reduction was observed in the IM group, where scores decreased from 120 to 54.5. Interestingly, the non-pharmacogenetically tested group also showed comparable improvement, from 120 to 54.5. The overall *p*-value for group differences was <0.001, indicating a highly significant association between genotype status and symptom reduction. These effects were more pronounced in patients who underwent regular MRI scans, particularly in the EM group. In contrast, IM patients with structural brain abnormalities exhibited slower and more variable response patterns.

For CGI-S, which measures clinical severity, all groups demonstrated significant improvement. Both EM and IM patients showed improvement, with a median score decreasing from 6 to 3, while the non-tested group decreased from 6 to 4. This pattern suggests that pharmacogenetic guidance may contribute to more consistent severity reduction, with a *p*-value of 0.003 confirming the significance of the intergroup differences.

CGI-I scores, evaluating perceived global improvement, followed a similar trend. All three groups converged to a median score of 2 at 18 months, starting from a median score of 4 at 1 month. The *p*-value of 0.007 supports the presence of statistically meaningful differences in how patients were perceived to respond to treatment over time.

Functional recovery, assessed via the GAF scale, also revealed distinct trajectories. The EM group improved from 39 to 76, achieving the highest functional levels at follow-up. The IM group progressed from 35.5 to 73, indicating significant recovery despite a lower baseline. In contrast, the non-tested group showed more modest gains, from 40 to 64. Again, the differences were statistically significant (*p* < 0.001), reinforcing the potential utility of genotype-informed treatment decisions in maximizing functional outcomes.

### 3.4. Analysis of Side Effects

The monitoring included a comprehensive assessment using the UKU Side Effect Rating Scale, the AIMS, and scales for extrapyramidal symptoms (SAS and BARS) (Table 5).

Across all scales, including UKU, AIMS, SAS, and BARS, the EM group demonstrated the most pronounced reduction in side-effect burden, with final scores substantially lower than baseline. The IM group also showed meaningful improvements, though residual side effects remained higher than in the EM group. The no pharmacogenetic testing group exhibited the least favorable reduction in side effects, with the highest residual scores across all four scales. Statistical analysis confirmed that the observed differences between groups were significant, with *p*-values ranging from 0.006 to 0.013.

In the following section, the analysis was further extended to include functional outcomes and depressive symptoms, as assessed by the Global Assessment of Functioning (GAF) and Calgary Depression Scale.

### 3.5. GAF and Calgary Depression Scale Outcomes

The GAF scale evaluates overall psychological, social, and occupational functioning, providing a composite measure of a patient’s functional status. At the same time, the Calgary Depression Scale assesses explicitly the severity of depressive symptoms in individuals with psychotic disorders.

Regarding functional outcomes, GAF scores increased substantially in all groups. Patients in the EM group achieved the highest functional recovery, with scores rising from 39 at baseline to 76 at 18 months. The IM group also showed notable improvement, with scores increasing from 35.5 to 73. The no-testing group experienced a more modest gain, from 40 to 64. The differences between groups were statistically significant (*p* < 0.001). In terms of depressive symptoms, as measured by the Calgary Depression Scale, all groups exhibited substantial reductions in scores over time. The EM group showed the most tremendous improvement, with scores decreasing from 8 to 2. The IM group improved from 9 to 3, while the no-testing group decreased from 10 to 4. The intergroup differences were statistically significant (*p* = 0.008). Both GAF scores and Calgary Depression Scale scores demonstrated substantial improvement across all genotype groups over the 18-month follow-up period (Table 6).

To account for the increased risk of Type I error due to multiple comparisons, a Bonferroni correction was applied to the primary outcome analyses. Given that seven independent comparisons were conducted across clinical scales (PANSS, GAF, Calgary Depression Scale, UKU, AIMS, SAS, and BARS), the significance threshold was adjusted from *p* < 0.05 to *p* < 0.0071. After correction, group differences in the PANSS, GAF, Calgary Depression Scale, and AIMS remained statistically significant. In contrast, differences observed in UKU, SAS, and BARS did not meet the corrected threshold for significance.

## 4. Discussion

This study investigated the relationship between CYP2D6 genetic polymorphisms, baseline neuroimaging findings, and clinical outcomes in a cohort of children and adolescents with psychotic disorders. The results support the initial hypothesis that integrating pharmacogenetic data and neuroimaging biomarkers can improve the precision of antipsychotic treatment and predict clinical evolution more reliably than either domain alone.

The analysis revealed that patients classified as EMs achieved the most favorable clinical outcomes, including greater reductions in psychotic symptoms (PANSS), improved global functioning (GAF), and fewer adverse effects (as measured by UKU, AIMS, SAS, and BARS). By contrast, IMs, particularly those with structural MRI abnormalities, displayed slower symptom remission and higher residual side-effect burden.

Statistical correlations confirmed that the CYP2D6 genotype significantly influenced both symptom trajectory and tolerability, while MRI findings added prognostic value, especially in identifying patients at risk of suboptimal outcomes. These findings underline the clinical utility of combined biomarker profiling for personalized psychiatric treatment in the pediatric population.

Findings from this investigation align with previous data reported in the literature; it is well documented that CYP2D6 polymorphisms significantly affect the metabolism of many atypical antipsychotics, altering both plasma drug levels and clinical efficacy [28,29,30,31]. Moreover, previous studies emphasize that in children and adolescents, the role of pharmacogenetics is even more critical, as the immature metabolic pathways of developing brains make them particularly vulnerable to adverse drug reactions [32,33,34,35,36].

The absence of PMs and UMs in this cohort limits the ability to evaluate the full spectrum of metabolic variability. However, the substantial proportion of IM patients underscores the clinical need for personalized dose adjustments to prevent subtherapeutic effects or toxicity. This is consistent with the findings from prior studies, which indicate that IM individuals often experience reduced treatment efficacy and increased risk of side effects unless dosing is carefully tailored [34,35]. The present study supports this recommendation, as pharmacogenetically guided therapy was associated with better clinical and functional outcomes, validating the utility of such an approach in routine clinical practice.

Particularly in developing pediatric brains, even subtle differences in metabolic capacity, such as those between EM and IM phenotypes, can influence both the efficacy and tolerability of antipsychotic treatment. Therefore, integrating CYP2D6 pharmacogenetic data into clinical decision-making is important to enhance safety and therapeutic precision, especially considering the high variability in enzyme activity observed in broader pediatric populations reported in the literature [37].

The results of the present study confirm that structural neuroimaging markers have prognostic value in children and adolescents with psychotic disorders. In this cohort, 20.63% of patients presented MRI abnormalities, most commonly brain cysts and sequelae of prior brain injury, with rare findings such as calcifications and venous angiomas also observed. Importantly, a strong correlation was found between CYP2D6 metabolizer status and the presence of MRI abnormalities, with IMs displaying a significantly higher prevalence of structural anomalies compared to EMs and non-tested patients. The absence of MRI abnormalities in the non-genotyped group was considered a coincidental finding, as all participants were enrolled according to the same clinical inclusion criteria, without any selection based on symptom intensity, risk level, or other differentiating factors. Prior neuroimaging studies in psychosis have consistently reported ventricular enlargement, cortical thinning, particularly in the frontal and temporal lobes, and volume reductions in subcortical regions such as the hippocampus and thalamus [38,39,40]. These findings have been linked to negative symptoms, cognitive dysfunction, and poor treatment response [8,41,42,43].

Taken together, these data support the utility of baseline MRI assessment not only for diagnostic clarification but also as a predictive tool for treatment planning, particularly when combined with genotypic profiling. Such integration may help identify high-risk profiles requiring closer monitoring and tailored therapeutic approaches.

The current study demonstrated a consistent and statistically significant reduction in PANSS total scores over the 18-month observation period across all patient groups, with the EM group showing the most pronounced and homogeneous improvement. This pattern reinforces the utility of pharmacogenetic guidance in enhancing symptom remission and supports the findings from the literature [44,45]. The CGI-S scores, which quantify illness severity, decreased significantly. This finding aligns with the existing literature, including large-scale validation studies of CGI-S in schizophrenia, which affirms its reliability in tracking severity changes during treatment [45]. Similar trends were observed in CGI-I, reflecting clinician-rated global improvement.

The results of the current study demonstrate a significant improvement in functional outcomes, as measured by the GAF scale, across all genotype groups during the 18-month follow-up period. The EM group achieved the highest gains in functionality (from 39 to 76), followed by the IM group (from 35.5 to 73) and the non-tested group (from 40 to 64). These findings align with the literature data, which highlights that functional recovery is an essential marker of long-term prognosis in pediatric psychosis [46,47]. Improvements in GAF scores in patients undergoing pharmacogenetically informed treatment underscore the role of personalized therapy in promoting better social, psychological, and occupational outcomes [48,49].

Regarding affective outcomes, as assessed by the Calgary Depression Scale, all groups exhibited a clear trend toward a reduction in depressive symptoms. The literature supports these findings that emphasize the Calgary scale’s specificity in detecting depression in psychotic patients, allowing the differentiation of actual depressive symptoms from negative symptoms or medication side effects [50,51]. Moreover, recent research referenced in the thesis indicates that improvements in depression scores are associated with enhanced resilience and quality of life in patients with psychotic disorders [51]. Interestingly, although Calgary score reductions were observed in all groups, statistical analyses revealed that these improvements were not significantly associated with genotype but rather reflected the general effectiveness of the integrated interventions applied in this cohort.

The present study confirmed that the CYP2D6 genotype influences the incidence and severity of antipsychotic-related side effects, with pharmacogenetically guided treatment improving tolerability. Patients in the EM group experienced fewer and less persistent side effects compared to the IM and non-tested groups, as reflected across multiple scales (UKU, AIMS, SAS, BARS) [52,53,54,55].

These findings are supported by the literature, which highlights the role of CYP2D6 polymorphisms in modulating adverse effects, including extrapyramidal symptoms, weight gain, and metabolic disturbances [56,57]. Variations in CYP2D6 activity influence drug plasma levels and the risk of toxicity, particularly with antipsychotics like Risperidone and Olanzapine, commonly used in pediatric populations [32,35]. Moreover, IM phenotypes are associated with an increased risk of adverse effects, including weight gain and hyperprolactinemia, which can negatively impact treatment adherence and quality of life [58,59,60]. This underscores the value of pre-emptive pharmacogenetic testing to tailor antipsychotic choice and dosing, particularly in children and adolescents [61,62]. The results of this study validate this approach, demonstrating that integrating genetic and clinical monitoring can significantly improve the safety profile of antipsychotic treatment in pediatric psychosis.

This study highlights the importance of early personalized interventions in pediatric psychosis; it supports associating such strategies with reduced severity of symptoms and better long-term prognosis [63,64,65]. The present results validate this view, as patients receiving pharmacogenetically guided treatment achieved faster and more consistent clinical improvement.

The strengths of this study lie in its integrated approach, which combines pharmacogenetic data with neuroimaging findings and comprehensive clinical assessments over an extended follow-up period, providing a robust basis for evaluating personalized antipsychotic treatment strategies in pediatric psychosis. However, certain limitations should be acknowledged.

One of the main limitations of this study is the use of structural MRI only, without incorporating functional imaging (fMRI) or magnetic resonance spectroscopy (MRS). At the same time, conventional MRI provided valuable anatomical insights, and advanced imaging techniques could have offered a more nuanced understanding of functional connectivity, neurochemical alterations, and brain activity patterns associated with treatment response. Additionally, the sample size and lack of PM or UM genotypes limit the generalizability of genotype-based conclusions across the full metabolic spectrum. This is because the non-tested group likely contains a mix of patients with varying CYP2D6 metabolizer statuses, which may have affected the accuracy of between-group comparisons. Although efforts were made to ensure sample comparability, potential confounding factors, such as socioeconomic background, comorbidities, and environmental influences, were not controlled and may have influenced the observed associations.

Future research should focus on larger, multicenter cohorts and aim to integrate multimodal neuroimaging with pharmacogenomic and clinical data to enhance understanding of the disease. Also, the subsequent research should consider integrating functional MRI and MR spectroscopy to complement anatomical findings and explore the neurochemical and connectivity patterns associated with treatment response. This approach could lead to the development of predictive algorithms for treatment response and tolerability, facilitating the creation of individualized treatment protocols. Further, longitudinal follow-up beyond 18 months could help elucidate the long-term impact of personalized interventions on cognitive function, social integration, and quality of life.

## 5. Conclusions

This study demonstrates that combining CYP2D6 pharmacogenetic profiling with neuroimaging biomarkers enhances the precision of antipsychotic treatment in children and adolescents with psychotic disorders. EMs showed the most significant improvements in symptom reduction (PANSS), global functioning (GAF), and side-effect profile (UKU, AIMS, SAS, BARS). In contrast, IMs, especially those with MRI abnormalities, had slower clinical recovery and more residual side effects. A strong correlation was found between CYP2D6 genotype and MRI findings, with IM patients more likely to exhibit structural brain anomalies. These combined biomarkers proved effective in predicting treatment response and guiding personalized interventions. The results support the early integration of genetic and imaging data into clinical practice to improve outcomes and reduce adverse effects in pediatric psychosis. Despite limitations, the findings underline the value of personalized psychiatry for vulnerable populations.

## Figures and Tables

**Table 1:** Distribution of metabolizer phenotypes among the patients.

CYP2D6 Metabolizer Phenotype	Number of Patients	Percentage (%)
No pharmacogenetic testing	32	50.8%
Poor Metabolizer (PM)	0	0%
Intermediate Metabolizer (IM)	12	19%
Extensive/Normal Metabolizer (EM)	19	30.2%
Ultra-rapid Metabolizer (UM)	0	0%

**Table 2:** MRI modification.

MRI Finding	Number of Patients	Percentage (%)
No modifications	50	79.37%
Sequelae of brain injuries	4	6.35%
Brain cysts	5	7.94%
Calcifications of the choroid plexuses and the pineal gland	1	1.59%
Magna cistern enlargement	2	3.17%
Venous angioma	1	1.59%

**Table 3:** The distribution of MRI findings among CYP2D6 metabolizer phenotypes.

Genotype	Normal MRI	Sequelae of Brain Injuries	Brain Cyst	Calcification	Magna Cistern Enlargement
EM + IM	32	0	0	0	0
EM	18	1	0	0	0
IM	0	3	5	1	2

EM = extensive metabolizer; IM = intermediate metabolizer; MRI = magnetic resonance imaging.

**Table 4:** Clinical evolution based on genotype.

Outcome		EM Group	IM Group	No Pharmacogenetic Testing Group	*p*-Value
PANSS Total Baseline → 18 months	Median	118 → 40	120 → 54.5	115.5 → 71	<0.001
	IQR interval	97.75–123.5 → 35.5–45	108.5–134.5 → 46–66	95–127 → 61–88.5	
	IQR	25.75 → 9.5	26 → 20	32 → 27.5	
CGI-S Baseline → 18 months	Median	6 → 3	6 → 3	6 → 4	0.003
	IQR interval	5–6 → 3- 3	6–6 →3–4	5.5–6 → 4–5	
	IQR	1 → 0	0 → 1	0.5 → 1	
CGI-I 1 month → 18 months	Median	4 → 2	4 → 2	4 → 2	0.007
	IQR interval	3–4 → 1.25–2	4–4 → 2–2	3–4 → 2–3	
	IQR	1 → 0.75	0 → 0	1 → 1	
GAF Baseline → 18 months	Median	39 → 76	35.5 → 73	40 → 64	<0.001
	IQR interval	35–45 → 75–77.75	33–41 → 68.5–75.5	35.5–46.5 → 55–69.5	
	IQR	10 → 2.75	8 → 7	11–14.5	

**Table 5:** Side-effect analysis.

Scale		EM Group	IM Group	No Pharmacogenetic Testing Group	*p*-Value
UKU Baseline → 18 months	Median	10 → 10	10 → 10	9 → 10	0.008
	IQR interval	9–11 → 8–11.75	9–12 → 8–12.5	7.5–11 → 8–12	
	IQR	2 → 3.75	3 → 4.5	3.5 → 4	
AIMS Baseline → 18 months	Median	8 → 2	9 → 4	10 → 5	0.006
	IQR interval	7–9 → 0.5–2.5	8–11 → 2–6	7–13 → 2–7	
	IQR	2 → 1	3 → 1	2 → 1	
SAS 1 month → 18 months	Median	0.1 → 0.1	0.15 → 0.1	0.1→ 0.1	0.011
	IQR interval	0.025–0.1 → 0–0.1	0.1–0.2 → 0.1–0.2	0.05–0.1 → 0.1–0.2	
	IQR	0.075 → 0.15	0.1 → 0.1	0.05 → 0.1	
BARS Baseline → 18 months	Median	4 → 0	4 → 0	10 → 2	0.015
	IQR interval	3–5 → 0–0.5	3–5 → 0–0.5	7–13 → 2–7	
	IQR	0 → 1	0 → 1	2 → 1	

**Table 6:** Functional outcomes and depressive symptoms.

Outcome		EM Group	IM Group	No Pharmacogenetic Testing Group	*p*-Value
GAF Baseline → 18 months	Median	39 → 76	35.5 → 73	40 → 64	<0.001
	IQR interval	35–45 → 47.25–67.75	30 -45 → 46–75	36–56.5 → 61.75–75.75	
	IQR	10 → 20.5	12 → 17.5	11.5 → 14	
Calgary Baseline → 18 months	Median	8 → 2	9 → 3	10 → 4	0.008
	IQR interval	0.5–16 → 0–4	0.5–16 → 0–4	4–17 → 2–6	
	IQR	1 → 0	4 → 1	4 → 1	

## Data Availability

The data supporting this study are available from the corresponding author upon request. However, due to ethical considerations, they are not publicly accessible.

## Abbreviations

The following abbreviations are used in this manuscript:

DSM-5	Diagnostic and Statistical Manual of Mental Disorders, Fifth Edition
CYP2D6	Cytochrome P450 2D6
MRI	Magnetic Resonance Imaging
RT-PCR	Reverse Transcription Polymerase Chain Reaction
CPIC	Clinical Pharmacogenetics Implementation Consortium
PharmGKB	Pharmacogenomics Knowledgebase
PANSS	Positive and Negative Syndrome Scale
CGI-S	Clinical Global Impression—Severity
CGI-I	Clinical Global Impression—Improvement
GAF	Global Assessment of Functioning
UKU	Udvalg for Kliniske Undersøgelser Side Effect Rating Scale
AIMS	Abnormal Involuntary Movement Scale
SAS	Simpson–Angus Scale
BARS	Barnes Akathisia Rating Scale
UM	Ultra-Rapid Metabolizer
EM	Extensive/Normal Metabolizer
IM	Intermediate Metabolizer
PM	Poor Metabolizer
fMRI	Functional Magnetic Resonance Imaging
MRS	Magnetic Resonance Spectroscopy
